# Unveiling the Molecular Basis of the Noonan Syndrome-Causing Mutation T42A of SHP2

**DOI:** 10.3390/ijms21020461

**Published:** 2020-01-10

**Authors:** Angelo Toto, Francesca Malagrinò, Lorenzo Visconti, Francesca Troilo, Stefano Gianni

**Affiliations:** Istituto Pasteur-Fondazione Cenci Bolognetti, Dipartimento di Scienze Biochimiche “A. Rossi Fanelli” and Istituto di Biologia e Patologia Molecolari del CNR, Sapienza Università di Roma, 00185 Rome, Italy; francescamalagrino@gmail.com (F.M.); lorenzo.visconti@uniroma1.it (L.V.); francesca.troilo@uniroma1.it (F.T.)

**Keywords:** protein binding, kinetics, mutagenesis

## Abstract

Noonan syndrome (NS) is a genetic disorder caused by the hyperactivation of the RAS-MAPK molecular pathway. About 50% of NS cases are caused by mutations affecting the SHP2 protein, a multi-domain phosphatase with a fundamental role in the regulation of the RAS-MAPK pathway. Most NS-causing mutations influence the stability of the inactive form of SHP2. However, one NS-causing mutation, namely T42A, occurs in the binding pocket of the N-SH2 domain of the protein. Here, we present a quantitative characterization of the effect of the T42A mutation on the binding of the N-terminal SH2 domain of SHP2 with a peptide mimicking Gab2, a fundamental interaction that triggers the activation of the phosphatase in the cellular environment. Our results show that whilst the T42A mutation does not affect the association rate constant with the ligand, it causes a dramatic increase of the affinity for Gab2. This effect is due to a remarkable decrease of the microscopic dissociation rate constant of over two orders of magnitudes. In an effort to investigate the molecular basis of the T42A mutation in causing Noonan syndrome, we also compare the experimental results with a more conservative variant, T42S. Our findings are discussed in the context of the structural data available on SHP2.

## 1. Introduction

Noonan syndrome (NS) is an autosomal dominant multisystem disorder with a prevalence of 1/1000–1/2500, characterized by cardiac defects, skeletal malformations, typical facial features, short stature, mental retardation, and cryptorchidism [1,2]. Cardiovascular defects are the most common feature of NS, present in about 90% of patients. Only Down syndrome exceeds NS in prevalence of congenital heart disease [3]. In the last few decades, NS has been extensively studied in order to understand the genetic causes of this disease. It has been discovered that the RAS-MAPK molecular pathway is hyper-activated in NS patients, and consequently several genes involved in its regulation have a role in the onset of NS, such as PTPN11, SOS1, RAF1, etc. [2,4,5,6]. Missense mutations affecting the PTPN11 gene are the major cause of NS, accounting for ~50% of the cases [7,8,9].

PTPN11 gene encodes for Src homology region 2-containing protein tyrosine phosphatase 2 (SHP2), a ubiquitous protein with a major role in the positive regulation of the RAS-MAPK pathway [10,11,12]. The three-dimensional structure of SHP2 is characterized by the presence of two SH2 domains (N-SH2 and C-SH2) located at the N-terminal portion of the protein, and a PTP domain, which exerts the catalytic functions [12]. SH2 domains have the role of recognizing and binding short specific portions of proteins characterized by the presence of a phosphor-tyrosine. The presence of SH2 domains allows SHP2 to interact with specific partners, such as Grb2-associated binder (Gab) family adapter proteins, allowing a correct signal transduction [11,13].

The catalytic activity of SHP2 is regulated by an auto-inhibition mechanism that involves the interaction between the N-SH2 and PTP domain. In the absence of binding, N-SH2 physically blocks the active site of the PTP domain, leading to the inhibition of its catalytic activity. On the contrary, when N-SH2 binds, a ligand undergoes a major structural rearrangement that activates the phosphatase [12]. A number of mutations affecting SHP2 have been connected with the onset of NS. Whilst the majority of these mutations concern residues at the interface between the N-SH2 and PTP domain (for example, D61G, A72S, A72G, E76D, I282V, N308D, M504V) [7,14,15] destabilizing the catalytically inactive form of SHP2 [16], one mutation, namely T42A, occurs in the N-SH2 domain far from the PTP domain. Interestingly, in the latter case, a comparison by ITC of the binding properties of wild-type and mutated N-SH2 revealed an increased capability of the naturally occurring variant; an observation which demands additional experiments.

In this communication, by characterizing the binding properties of the T42A variant in comparison to that of wild-type N-SH2, we investigate the molecular basis by which the mutation leads to the disease. In particular, by following the change in fluorescence signal due to Förster resonance energy transfer between a tryptophan residue naturally present in the N-SH2 domain and a dansyl group covalently attached to the peptide mimicking Gab2, a physiological ligand of SHP2, we could measure the microscopic association and dissociation rate constants of binding. The comparison of kinetic data obtained with wild-type and mutated N-SH2 revealed a dramatic increase in the affinity for Gab2 in the mutant, due to a pronounced decrease of the microscopic dissociation rate constant. Our data are briefly discussed on the basis of available structural data and previous works on the T42A NS-causing mutation.

## 2. Results

**Figure 1:**
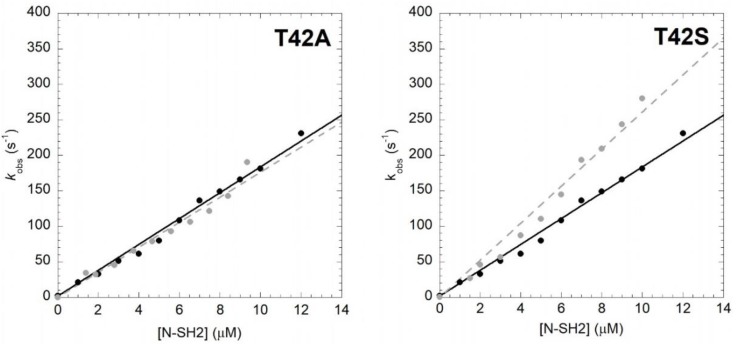
Kinetics of binding between Gab2_608−620_ and N-SH2 wt (black circles, black continuous line) and its variants (gray circles, gray broken lines) in buffer Hepes 50 mM pH 7.2, NaCl 300 mM, at 10 °C. wt data were taken from [17]. Lines are the best fit to a linear equation.

In order to unveil the effect of T42A mutation on the binding properties of the N-SH2 domain, we resorted to performing kinetic binding experiments. In analogy to our previous work [17], the binding between N-SH2 and Gab2 was measured by using a dansylated peptide mimicking the region of Gab2 ranging between residues 608 and 620 (Gab2_608−620_), presenting a phospho-tyrosine in position 614, which is specifically recognized by N-SH2. The binding reaction was measured by stopped-flow mixing experiments, by following the change in FRET signal occurring between residue tryptophan 6 of N-SH2 and a dansyl group covalently linked to the N-terminus of Gab2_608−620_. Pseudo-first-order binding experiments were performed by rapidly mixing a constant concentration of dansylated Gab2_608−620_ (1 μM) versus different concentrations of N-SH2 T42A, ranging from 1 to 12 μM. All traces followed a single exponential decay. A comparison between the observed rate constants obtained for N-SH2 T42A to those of the wild-type proteins at the same experimental conditions is reported in Figure 1. Both experiments were consistent with a linear dependence of pseudo-first-order kinetics. It is evident that the T42A mutation has no effect on the recognition event of Gab2_608−620_, the calculated *k*_on_ values (*k*_on_ T42A = 17.6 ± 0.6 μM^−1^ s^−1^) being very similar to the one obtained for N-SH2 wt (*k*_on_ wt = 18.2 ± 0.3 μM^−1^ s^−1^).

**Figure 2:**
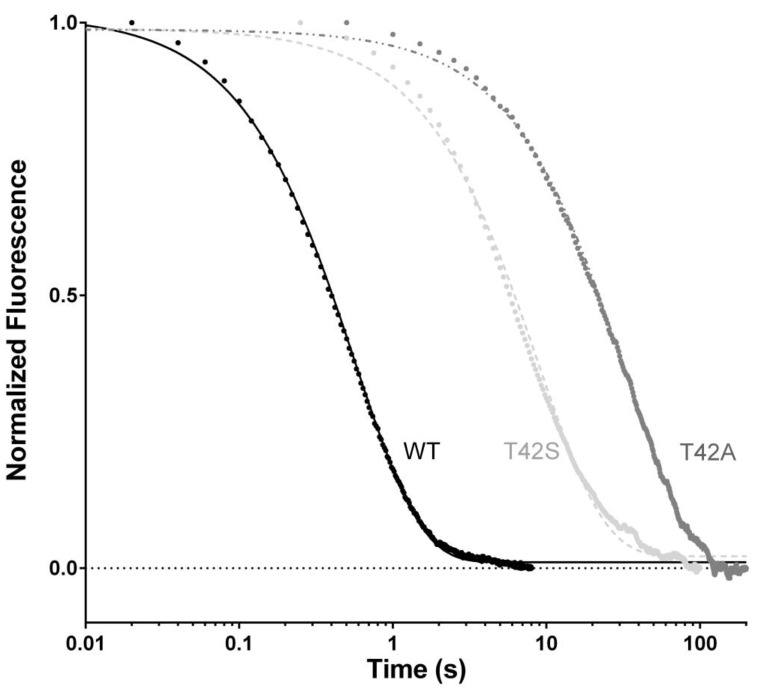
Displacement of the complex N-SH2:dansyl-Gab2_608−620_ by a large excess of non-dansylated Gab2_608−620_. Lines represent the best fit to a single exponential equation.

In theory, analysis of the dependence of the observed rate constants under pseudo-first-order conditions would allow the microscopic dissociation rate constants (*k*_off_) to be calculated by extrapolating at 0 [N-SH2]. However, because of the low values of *k*_off_, the high experimental error that arises from this procedure jeopardizes a valuable calculation of *k*_off_ and, therefore, demands a different approach. To accurately determine *k*_off_ values, we resorted to performing displacement experiments, in which a pre-incubated complex of dansylated Gab2_608−620_ and N-SH2 (at 1:1 stoichiometric ratio) were rapidly mixed with different concentrations, in high excess, of nondansylated Gab2_608−620_. In agreement with theory [18], when the concentration of the displacer was in high excess compared to that of the pre-incubated complex, the observed rate constant was found to be insensitive to reactant concentrations. The displacement time courses for N-SH2 T42A and wild-type N-SH2 are reported in Figure 2. In both cases, data were satisfactorily fitted with a single exponential equation. Interestingly, the T42A mutation caused a remarkable effect on *k*_off_, being 100 times lower compared to the one obtained for wild-type N-SH2 under the same experimental conditions (*k*_off_ wt = 1.85 ± 0.10 s^−1^; *k*_off_ T42A = 0.031 ± 0.005 s^−1^). The direct measurement of *k*_off_ allowed us to obtain the equilibrium dissociation rate constant ($K_{D}=\;k_{\mathrm{off}}/k_{\mathrm{on}}$) for the interaction between N-SH2 T42A and Gab2. The affinity for the ligand was dramatically increased upon mutation, with a *K*_D_ = 1.7 ± 0.1 nM calculated for N-SH2 T42A compared to the *K*_D_ = 100 ± 10 nM obtained under the same experimental conditions for wild type N-SH2.

In order to obtain more detailed information about the role of T42 residue in the binding reaction with Gab2, we designed a more conservative N-SH2 variant where the threonine was replaced with a serine. This variant retains the hydroxyl group while removing an electron donor methyl group from the side chain. In analogy to the T42A mutant, we performed binding (Figure 1) and displacement (Figure 2) kinetic experiments on the T42S mutant and compared them to those of the wild-type protein. The *k*_on_ value calculated from fitting was comparable with the one obtained for the binding with N-SH2 wt (*k*_on_ T42S = 26.1 ± 0.7 μM^−1^ s^−1^), indicating that the T42S mutation also does not affect the recognition of Gab2_608−620_. On the other hand, the value of *k*_off_ appears to be affected by T42S mutation by a factor of 10 compared to that of the wild type, with a calculated value of 0.14 ± 0.02 s^−1^, and consequently an equilibrium dissociation constant of 5.3 ± 0.2 nM.

**Figure 3:**
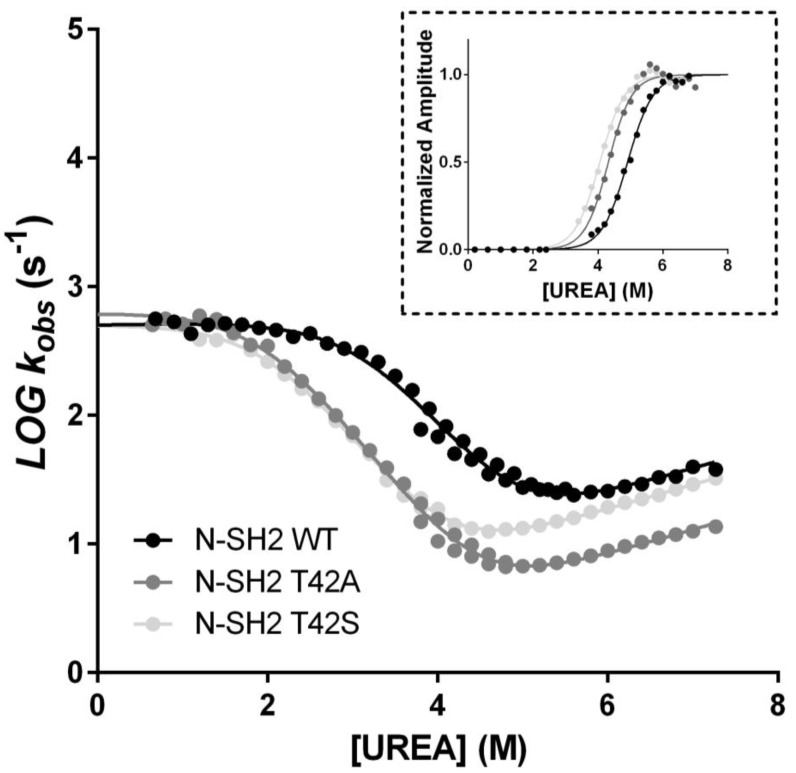
Plot of the log_10_ of the (un)folding observed rate constants measured at different urea concentrations for N-SH2 WT (black circles), N-SH2 T42A (dark gray circles), and N-SH2 T42S (light gray circles) measured at the stopped-flow apparatus in buffer Tris-HCl 50 mM, pH 8.0, at 25 °C. Lines represent the best fit to an equation describing a three-state folding mechanism (see text for details). Inset panel: analysis of the amplitudes obtained in unfolding experiments as a function of urea concentration. Lines are the best fit to a sigmoid equation. It is evident that the mutations contribute to a minor destabilization without significantly altering the folding of N-SH2.

To test the effects of the T42S and T42A mutations of the structure of N-SH2, we carried out a complete folding characterization of these two variants, in comparison to the wild-type protein. In particular, we subjected the mutants to equilibrium and kinetic unfolding experiments. The experiments were carried out following the same procedures described previously for N-SH2 [17,19]. The folding and unfolding data obtained for T42S and T42A in comparison to wild-type N-SH2 are reported in Figure 3. It is evident that, whilst the T42S mutant contributes to a minor destabilization of the native state, in both cases the folding pathway is essentially unaffected by the mutations, as proved by the apparent co-operativity of the equilibrium transitions as well as by the dependence of the folding and unfolding rate constants. These findings suggest that the mutations do not significantly alter the folding of N-SH2.

## 3. Discussion

The collaborative efforts between structural biology and enzymatic essays on SHP2 has provided compelling evidence in favor of an auto-inhibition model at the basis of the regulation of this critical enzyme [12,20]. In this scenario, the N-SH2 domain has been depicted as a conformational switch—either it blocks the catalytic activity of the PTP or, when bound to a pY-containing sequence, it releases the PTP domain, thereby tuning its activity. In this context, it is of particular interest to characterize the only NS-causing mutation that does not occur at the interface between the N-SH2 and PTP domain, T42A. Phosphatase assays conducted on SHP2 protein [20] revealed that T42A SHP2 displays a dramatically enhanced activity when in the presence of a phospho-tyrosine containing peptide, compared to wild-type SHP2 and other variants not associated with NS, whilst its basal activity was comparable with the wild-type form. It has also been demonstrated that T42A mutation causes an impairment of the binding capabilities of SHP2 [21], however, no information is available about the details of this mutation’s effect on the interaction of the enzyme with its natural binding partners. In this work, we provide a kinetic characterization of the effects of the NS-causing T42A mutation on the binding of the N-SH2 domain with a peptide mimicking the scaffolding protein Gab2, an interaction naturally occurring in the cellular environment that triggers the activation of SHP2. Interestingly, while the mutation has no effect on the recognition of the ligand, with a conserved value of *k*_on_, the complex appears to be strongly stabilized upon T42A mutation. In fact, the dramatic effect observed on *k*_off_ demonstrates that T42A mutation causes a less efficient release of the ligand from the N-SH2. Thus, in agreement with the auto-inhibition model, on the basis of our data we conclude that T42A mutation limits the ability of N-SH2 to act as a conformational switch, promoting the activated form of SHP2 at lower ligand concentrations.

**Figure 4:**
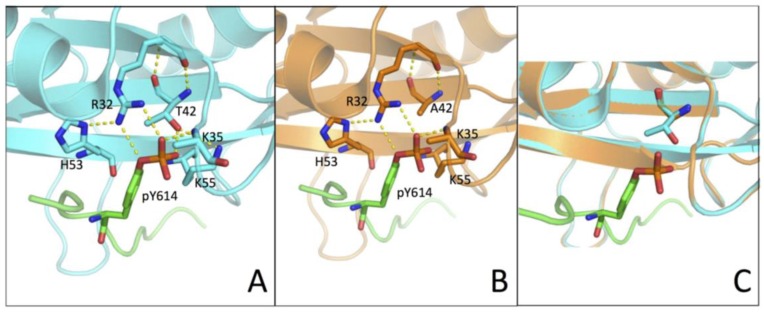
(**A**,**B**) Three-dimensional structure of the N-SH2 domain wt (in cyan) and T42A (in orange) in complex with Gab1 (in green) (PDB: 4QSY). Residues R32, K35, T42/A42, H53, and K55 are highlighted with sticks and H-bonds are represented by yellow broken lines. (**C**) Homology modelling of the T42A variant of the N-SH2 domain (in orange) computed with SWISS-MODEL web-based service, using PDB 4QSY as template structure (in cyan). Side-chains of T42/A42 are highlighted with sticks.

It is of interest to analyze our kinetic data in the light of the three-dimensional structure of N-SH2 (PDB: 4QSY). In fact, the structure shows that the hydroxyl group of T42 residue is in direct contact with the phosphate group of the phospho-tyrosine (Figure 4). Our results showed how a more conservative mutation, T42S, which maintains the polar properties of the side chain while removing an electron-donor methyl group, causes a less pronounced effect on the binding affinity. Of additional interest, it may be noted that previous work based on molecular dynamics highlighted the presence of a conformational switch in N-SH2, primarily mediated by Tyr66, Asp40, Lys55, and Gln57 [22]. Importantly, out of these residues, Lys55, lies in the proximity of T42. On the basis of these observations, we conclude that the hydroxyl group of T42 can have a role in shielding the negative charges of the phospho-tyrosine, as well as in mediating the role of Lys55 in an opening-closing motion associated with ligand binding, thereby tuning the physiologically correct rate of release of the ligand. Taken together, our results suggest that the T42A mutation found in NS patients impairs this mechanism of ligand release and consequently promotes a hyper-activated form of SHP2, which is the basis of the onset of the disease.

## 4. Materials and Methods

### 4.1. Protein Expression and Purification 

N-SH2 wild-type and the site-directed variants T42A and T42S were expressed and purified as described previously [17]. The variants were obtained using a QuickChange Lightning Site-Directed Mutagenesis kit (Agilent Technologies, Santa Clara, California, United States), accordingly to the manufacturer’s instructions.

### 4.2. Stopped-Flow Experiments 

Binding kinetics experiments were performed using a single-mixing SX-18 stopped-flow instrument (Applied Photophysics, Leatherhead, Surrey, UK,); binding experiments were conducted in buffer Hepes 50 mM pH 7.2, NaCl 300 mM, at 10 °C. Pseudo-first-order binding experiments were performed by mixing a constant concentration of dansylated Gab2_608−620_ (1 μM) versus N-SH2 wt and its variants at concentrations ranging from 1.5 μM to 12 μM. Displacement experiments were performed by mixing the pre-incubated complex N-SH2:Gab2_608−620_ at the concentration of 1 μM with the non-dansylated form of Gab2_608−620_ at increasing concentrations, ranging from 30 μM to 60 μM. The excitation wavelength for all the experiments was 280 nm, and emission was collected using a 475-nm cut-off glass filter. For each acquisition, the average of at least five independent experiments was fitted to a single exponential equation. Kinetic traces are provided as supplemental information (Figure S1).

Unfolding and refolding kinetic experiments were conducted using a single-mixing SX-18 stopped-flow. Excitation wavelength was 280 nm and change in tryptophan fluorescence emission was followed by using a 320 nm cut-off filter. Experiments were performed in buffer Tris-HCl 50 mM, pH 8.0, at 25 °C. Protein concentration was 3 μM.

### 4.3. Structure Homology Modelling

The three-dimensional structure of the T42A variant of the N-SH2 domain was computed with SWISS-MODEL web-based service (https://swissmodel.expasy.org/), by using PDB 4QSY as the template structure.

## Supplementary Materials

Supplementary materials can be found at https://www.mdpi.com/1422-0067/21/2/461/s1.
