# Supplementary material for: Unveiling the Molecular Basis of the Noonan Syndrome-Causing Mutation T42A of SHP2
Source: Int J Mol Sci. 2020 Jan 10;21(2):461. doi: 10.3390/ijms21020461 (PMC7013464; doi:10.3390/ijms21020461)
Supplement: Supplementary file 1 [file ijms-21-00461-s001.pdf]

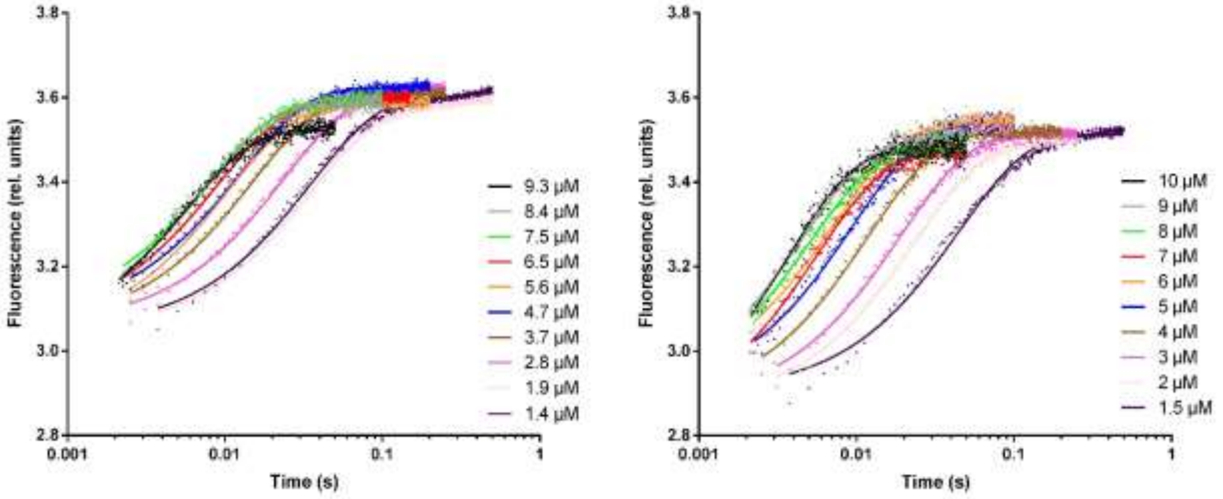

Figure S1. – Kinetic traces obtained by challenging a constant concentration Dansyl-Gab2$_{608\text{–}620}$ (1 µM) versus different concentrations of N-SH2 T42A (left panel) and N-SH2 T42S (right panel) in stopped-flow binding experiments. Experiments were conducted in buffer Tris-HCl 50 mM, pH 8.0, NaCl 300 mM, at 10 °C. Lines are the best fit to a single exponential equation.
